# High-Throughput Microfluidic Technologies for Rapidly Screening Pollutant-Induced Cell Health Effects

**DOI:** 10.1021/acsbiomedchemau.5c00094

**Published:** 2025-08-06

**Authors:** Blanca I. Quiñones-Díaz, Niphattha Wongwiset, Pratik Kamat, Orian Stapleton, Sean M. Engels, Matthew R. Burroughs, S. V. Sreenivasan, Jude M. Phillip, Lydia M. Contreras

**Affiliations:** † McKetta Department of Chemical Engineering, 12330 University of Texas at Austin, Austin, Texas 78712, United States; ‡ Department of Mechanical Engineering, University of Texas at Austin, Austin, Texas 78712, United States; § Department of Chemical and Biomolecular Engineering, 1466 Johns Hopkins University, Baltimore, Maryland 21218, United States; ∥ Department of Biomedical Engineering, Johns Hopkins University, Baltimore, Maryland 21218, United States; ⊥ Department of Oncology, Sidney Kimmel Comprehensive Cancer Center, Johns Hopkins University, Baltimore, Maryland 21287, United States; # Translational Tissue Engineering Center (TTEC), Johns Hopkins University, Baltimore, Maryland 21287, United States; ¶ Institute for Nanobiotechnology (INBT), Johns Hopkins University, Baltimore, Maryland 21218, United States

**Keywords:** microfluidic, morphology, pollution, lung cells, particulate matter

## Abstract

Air pollution exposure is linked to diseases spanning multiple physiological systems. However, environmental stress is overwhelmingly associated with several lung diseases. Since the chemical composition of air pollutants varies widely across geographical locations, research on how specific components in pollutant mixtures contribute to cellular dysfunction is needed. In this work, we utilized microscopy-based morphological profiling as a tool to assess the cellular susceptibility to pollutants. Through our analysis, we established morphological profiles of formaldehyde-exposed cells that showed dose-dependent shifts in cell shape profiles correlating with overall cell health. As a real-world proof-of-concept validation, we evaluated the differences in particulate matter (PM) composition across multiple geographical areas, including both urban and suburban communities in Austin, Texas, USA. Data from this real-world study was used to inform a multicombinatorial study involving metals, selenium (Se) and manganese (Mn), which were differentially abundant across PM collection sites. As proof of concept to demonstrate the potential of establishing low-cost, high-throughput combinatorial screening of the biological effects of these metals, we incorporated microfluidic technology to simultaneously generate variable two-component metal mixtures in a multiwell plate format that enabled microscopy-based morphological screening as a proxy for toxicity. Combinatorial analysis of Se and Mn showed dynamic cell responses across a range of concentrations. Interestingly, exposure mixtures containing both Se and Mn yielded healthier cellular phenotypes relative to Se exposure as a single component. These results demonstrate the development of a high-throughput pipeline to detect dynamic biological responses to common air pollutants. Leveraging multiple technologies, we demonstrate the feasibility of a cost-effective approach that can serve as a starting point to inform focused screenings and studies of air pollutant mixtures that affect health outcomes.

## Graphical abstract

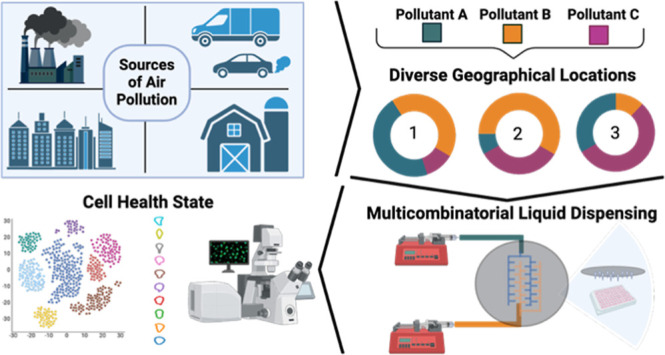

## Introduction

Air pollution represents an increasing concern to human health, with millions of deaths annually being attributed to environmental exposure to pollutants.^1^ One of the most common sources of air pollution exposure is particulate matter (PM). PM represents a mixture of liquid droplets and solid particles that vary in their chemical composition and biophysical properties. PM particles range in size, with particles of less than 2.5 μm (PM2.5) classified as fine PM and particles between 2.5 and 10 μm (PM10) classified as coarse PM.^2^ Both classifications have been associated with various adverse health effects, including cardiovascular, respiratory, and neurological diseases.^3–5^ However, PM2.5 has been demonstrated to penetrate deeper into the lung tissue and have elevated deposition in the epithelium, making it especially detrimental to human health.^6,7^

PM is known to vary greatly across geographical areas, predominantly arising from different human activities (e.g., agriculture, construction, industrialization, transportation, coal/fossil fuel burning for power plants, etc.).^8–10^ Previous research has shown that the biological response to PM exposure is heavily dependent on its composition.^11–14^ Particularly, we have previously observed that lung cells exhibit differential molecular and biophysical phenotypes in response to urban, fine, and diesel PM samples.^11^ Additionally, we have shown that these cellular responses can be driven, in part, by the differences in metal composition (e.g., cadmium and lead). Addressing these differences in pollution-driven health impacts requires the implementation of new strategies to systematically evaluate air pollution content across diverse geographical areas and study how these unique compositional differences influence health outcomes of various communities.

Several high-throughput technologies in the biological sciences have emerged in the past decade as useful tools to bypass many of the time, cost, and accuracy shortcomings inherent to complex laboratory processes.^15^ For biological applications, mass spectrometry, nucleic acid sequencing, fluorescence assays, chromatography, among other technological advancements, have been successfully adapted to high-throughput platforms through the implementation of liquid-handling devices and automated sampling and analysis pipelines.^16–18^ Research areas like drug discovery have been positively impacted by the technological advancements in high-throughput screening (HTS) methodologies, which can produce cellular responses for thousands of compounds in a single day.^19^ Despite the significant advances that high-throughput technologies provide, there remain underexplored fields that could benefit from the implementation of these technologies. In addition, most of these platforms are very expensive and not accessible to all researchers (particularly those in low-resource settings), which further highlights the need to propose new technologies that could provide cost-effective solutions. The design of microfluidic devices with multiple dispensing capabilities has enabled the development of rapid and cost-effective technologies for drug screening, nutrient delivery, and a variety of biochemical assays.^20,21^ Furthermore, toxicity assessment has been traditionally performed utilizing assays that indirectly measure the viability of the cells through markers of metabolic activity, membrane integrity, immunological responses, or oxidative stress.^22^ Recently, we have also demonstrated that tracking morphological parameters via microscopy techniques can accurately describe the health and responses of model epithelial lung cells to pollutant-induced stress.^11,23^

In this work, we developed a high-throughput pipeline that engages microfluidics coupled to microscopy techniques to understand the direct impact of individual air pollution components and multicomponent mixtures on cell physiology. Specifically, we constructed a novel microfluidic device for generating and dispensing various air pollution mixtures, enabling rapid screening via microscopy-based morphological profiling as a high-throughput cell response output. We validated the performance of the newly established microfluidic device by assessing its accuracy and precision in dispensing liquid dye into multiwell plates. Upon establishing the ability of the microfluidic device to formulate various mixtures in multiwell plates, we developed a morphology-based analysis workflow by coupling microscopy techniques to characterize morphological response profiles of BEAS-2B model human epithelial lung cells. From this analysis, first established using formaldehyde exposure, we learned that morphological profiling can sensitively detect responses to stress across a varied range of exposure concentrations. Subsequently, we investigated real-world PM samples by analyzing the elemental composition of PM samples obtained within different geographical locations in Austin, Texas, USA. As a proof of concept, we partially modeled these complex mixtures using the microfluidic device with two components identified as highly variable from the characterized PM samples, selenium (Se) and manganese (Mn). Interestingly, morphological profiling of Mn and Se mixtures showed that the combination of these components elicits a differential morphological response relative to that of single components alone in a dose-dependent manner. Lastly, we showed that single components and mixtures modulate emergent cellular heterogeneity, with a tendency toward low cellular heterogeneity for cells exposed to higher doses of Se.

## Results

### Morphology Is a Sensitive Indicator of Pollutant-Induced Lung Cell Health

In prior work, we showed that exposure to different PM mixtures leads to differential cellular response at both the molecular and morphological levels across cell populations.^11^ Morphology has been closely linked to damaged cell states, such as apoptotic and senescence processes that normally occur in response to stress conditions.^24,25^ Moreover, in previous work, we demonstrated that specific morphological states driven by exposure to PM highly correlate with DNA damage markers and adverse cell health.^11^ Given its generality and strong association with cell health states, morphological profiling provides a sensitive tool to describe the toxicity of air pollutants on human cells. To develop and train our morphological pipeline and validate its potential use as a biomarker of cell health, we exposed cells to liquid formaldehyde since we previously observed morphological changes after exposure to gaseous formaldehyde in human lung cell models.^26^

To this end, we treated human model BEAS-2B lung cells with serial dilutions of formaldehyde in cell culture media (31–500 μM) and imaged cells after 24 h of exposure. We subsequently extracted the morphological data from these images using CellProfiler and performed dimensional reduction. We visualized the results on a two-dimensional uniform manifold approximation and projection (UMAP) map to reduce 32 morphological parameters into two dimensions.^27^ Using K-means clustering, we identified 8 clusters with distinct morphological features ranging from larger, elongated cells to smaller, spherical cells (Figure 1A). When projecting the concentration gradient of formaldehyde exposures onto the UMAP manifold, we observed a clear, dose-dependent transition in cell morphology from healthy, elongated, large cells (nontreated) to unhealthy, spherical, smaller cells (high formaldehyde concentration) (Supporting Information Figure S1A). Confirming this trend, we performed quantitative analysis of nuclear and cell area, showing that clusters 1–2 (left side of the manifold) are the smallest, clusters 7–8 (top of the manifold) are the largest, and clusters 3–6 show intermediate size values (Figures 1B,C and Supporting Information S1B). To delineate the association of these morphologies to cellular phenotypes that were compromised by formaldehyde exposures, we utilized Calcein, a chemical compound that fluoresces when hydrolyzed by cellular esterases, as a proxy for cell viability. Indeed, the Calcein intensity values were higher in clusters 6–8 and lower in clusters 1–5 (Figure 1D). Further demonstration of this correlation was observed through direct quantification of cluster enrichment across the different levels of formaldehyde exposure (Figure 1E). Nontreated cells were enriched in cluster 6, whereas slightly elevated concentrations (31 and 62 μM) of formaldehyde resulted in population shifts toward clusters 7–8. Interestingly, 125 and 250 μM formaldehyde exposures presented an almost uniform enrichment in clusters 3 and 5, while the higher 500 μM exposure was significantly enriched in cluster 5 alone. While we start to observe morphological shifts at 31 μM formaldehyde exposures, bulk viability assays do not show any significant difference until 250 μM formaldehyde (Supporting Information Figure S2). Overall, these results highlight the sensitivity of our morphological profiling pipeline to identify intermediate transitional cellular states in response to stress that are not captured by traditional toxicological assays.

**Figure 1:**
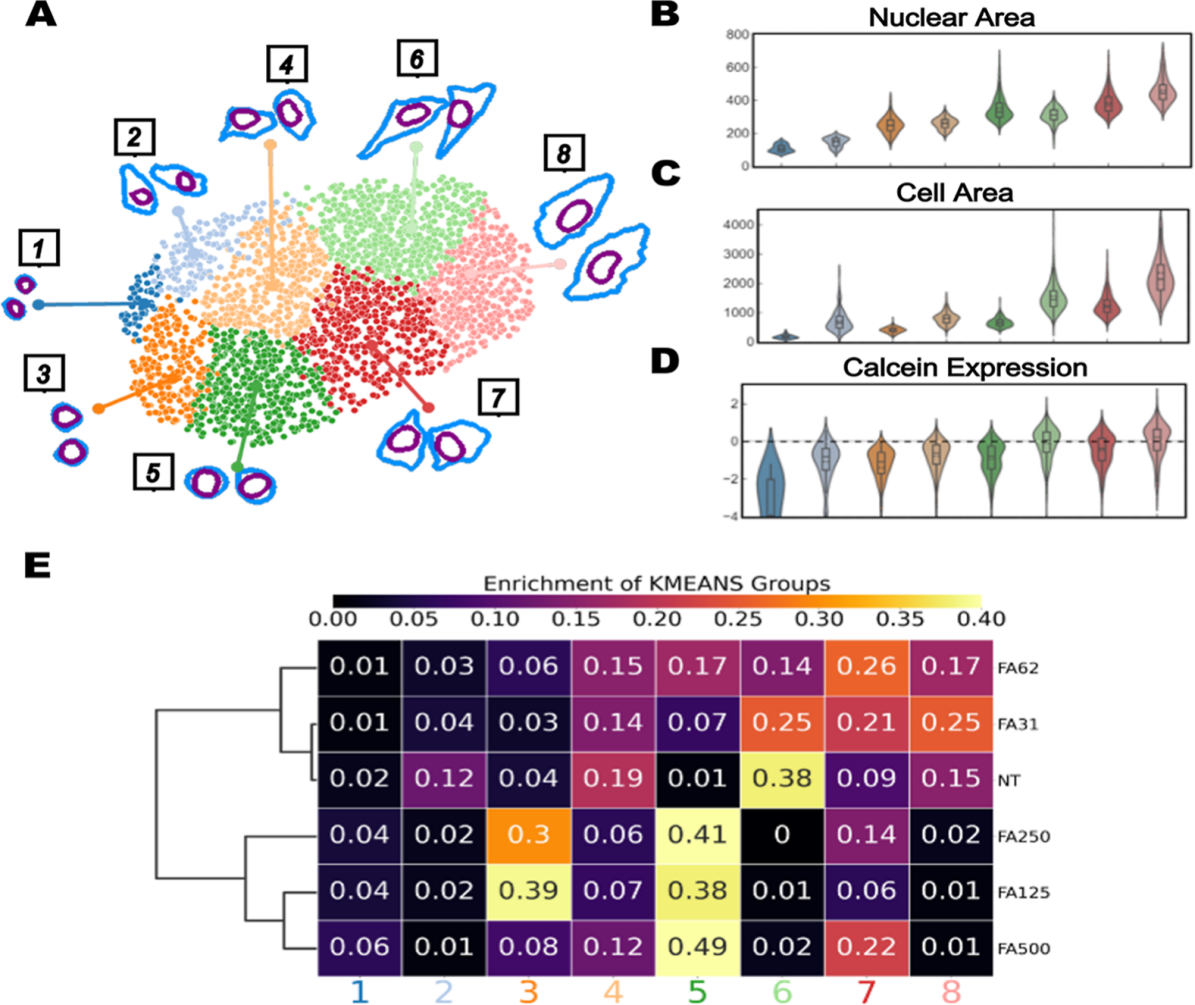
Morphological profiling of pollutant-exposed cells describes differential cell states. (A) UMAP 2-D representation of the reduction of 32 morphological parameters for nontreated cells and formaldehyde exposures ranging from 31 to 500 μM. Cell feature quantification of (B) nuclear area, (C) cell area, and (D) calcein expression (Z-score) for all clusters. (E) Heatmap of cluster enrichment by the exposure condition.

### Compositional Characterization of PM Mixtures from Geographically Diverse Sites Indicates Se and Mn as Highly Variable Metal Components

Given that ambient air pollution is a complex, multicomponent mixture of compounds that varies widely depending on geographic location,^28^ we next sought to investigate the versatility of our approach toward assessing real-world air pollution compositions. To accomplish this goal, we characterized the elemental diversity of particulate matter across four different locations in Austin, Texas, USA (Figure 2A). These locations were selected based on geographical and air pollution monitoring data (i.e., black carbon levels) to capture areas within Austin that represent different levels and compositions of air pollution exposure (Figure 2B).^29^ Particularly, locations A and B were in predominantly urban areas less than 2 miles away from major highways or high-traffic roads, whereas locations C and D were in more suburban neighborhoods near green areas (i.e., parks).

**Figure 2:**
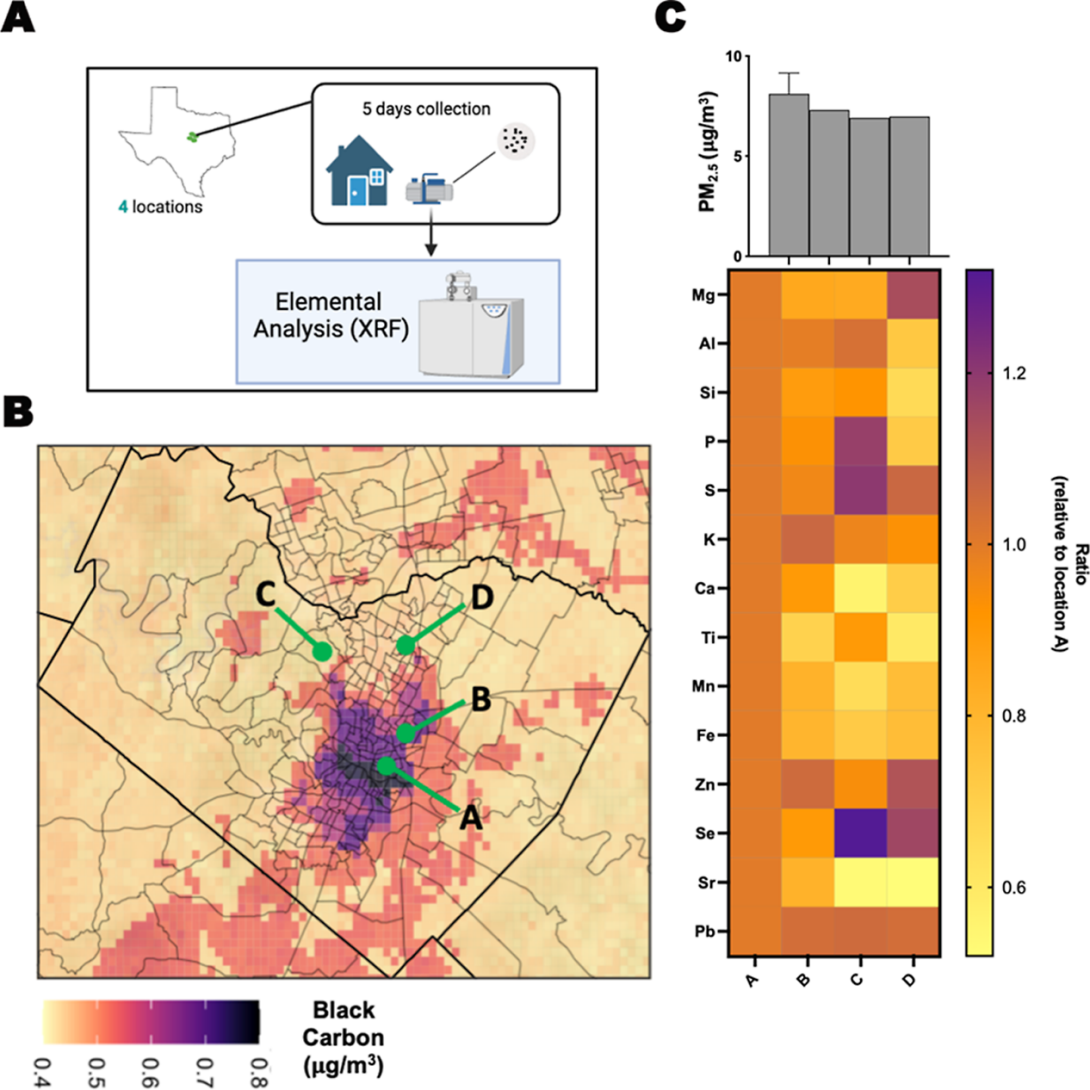
Compositional differences in PM samples from different geographical areas. (A) Scheme of PM collection and analysis process. (B) Black carbon monitoring map of Austin, TX, highlighting sampled locations. (C) Abundance ratios of quantified elements calculated relative to location A. The top panel reflects the total PM concentration.

Across the four locations, the total PM2.5 concentration was of relatively equal magnitude; a slightly elevated average concentration was collected on locations A and B relative to locations C and D (Figure 2C). These elevated concentrations were expected, given the relative proximity of these sites to urban centers. To evaluate compositions of these PM samples, we conducted elemental composition analysis, which yielded quantifiable data for 14 elements (Supporting Information Table S1). Using location A as a reference normalization control, we observed differential levels (±20%) of P, S, Ca, Ti, Mn, Se, Sr, and Si in at least one location relative to location A (Figure 2C). As expected by the black carbon maps and geographical proximity, locations A and B had the most similar elemental profiles of all locations. In contrast, location C, which is located in the area with the least black carbon, had higher levels of P, S, and Se and lower levels of Ca, Mn, and Sr. Location D exhibited similar trends to location C, but in a lesser magnitude and with distinct levels of Mg. Although this analysis was performed with a small sample size (*N* = 4 locations), it demonstrates the influence of geographic location on the composition of ambient air pollution and further showcases the need to evaluate how different mixtures of pollutants drive differential cellular responses that could be linked to health outcomes. Furthermore, this analysis identified manganese (Mn) and selenium (Se) as two highly variable metals across locations that we hypothesized could differentially impact cell viability and pollutant susceptibility.

### Incorporation of Microfluidic Technology for Multicomponent Parallel Dispensing to Allow Rapid Generation of Varying Pollutant Mixtures

Microscopy is a highly versatile experimental tool that can be readily adapted for high-throughput approaches by using multiwell plates and rapid-automatable image collection programs. However, the process of manually performing multiplexed component exposures is a time-consuming process that is highly prone to user error. To circumvent this potential obstacle when evaluating multiple pollutants in a systematic format, we employed a parallel dispensation approach with a microfluidic device that can simultaneously provide combinatorial component concentrations. The device was fabricated as described in the Methods section, and the experimental setting is shown in Figure 3A,B.

**Figure 3:**
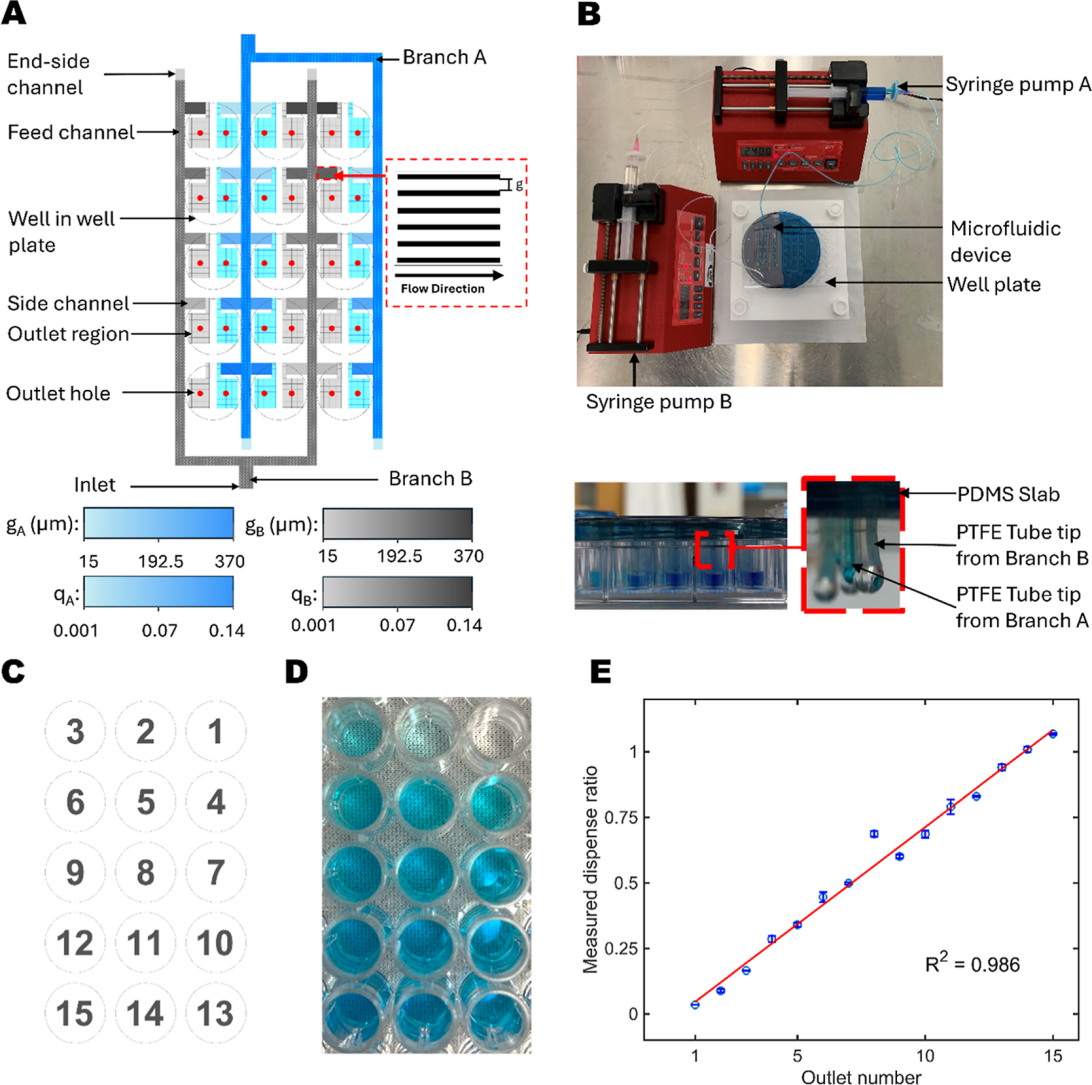
Microfluidic device design for multicombinatorial dispensing. (A) Schematic of the microfluidic device consists of 2 branches (A,B) to infuse 2 liquids. Parts within each branch labeled within branch B include the inlet, feed channel, side channels, end-side channels, outlet regions, and outlet holes. The inset shows a side-channel structure, which is a parallel rectangular channel. The gradients at the bottom represent various side-channel gaps (g_A_ and g_B_) and relative outlet flow rates with respect to an inlet flow rate (q_A_ and q_B_) for branches A and B, respectively. (B) Overview of the experimental setting in top and side views. (C) Reference schematic of outlet numbers of the microfluidic device. (D) Picture of generated combinatorial concentrations by infusing dye and DI water into branches A and B, respectively. (E) Concentration plot with respect to the label location, where a red line is a linear trendline fitting. The experiment was performed in three replicates. Nominal values and uncertainties represent averages and standard deviations.

Within the device, two liquids were loaded into the channels using syringe pumps, and each liquid separately flowed into an inlet branch (branches A and B). The solution syringes had 0.45 μm filters to reduce debris and undesirable contamination injected into the device. The liquid within each branch is then distributed through the feed channel and spread to the smaller branches near the outlets (side channels). All side channels are parallel rectangles with a constant pitch and varied gaps. These varied gaps allowed different amounts of liquids to pass through the side channels; a channel with a larger gap possesses less fluid resistance so that more liquid could pass through, as shown in Figure 3A. To vent bubbles out of the device while minimizing solution waste, the end-side channels, which had more resistance than side-channel resistances, were designed and located at the end of the feed channel. The flow ratio design of the device is further explained in Supporting Information. At outlets, we connected PTFE tube tips so that the liquid could be more easily dispensed due to the PTFE hydrophobicity. The inlet and outlet tubes were secured in place by PDMS connector slabs, as shown in Figure 3B.

The microfluidic device was able to simultaneously provide 15 linear-profile concentrations into a 96-well plate in 3 min. The dispensing ratios between the two solutions were evaluated by measuring the food-dye concentrations at the outlets where dye and deionized (DI) water were infused into branches A and B, respectively. The concentrations were calculated based on the reference intensity of standard concentrations measured by a plate reader. Figure 3C–E shows that the device created linear concentrations with *r* ^2^ = 0.986 from a linear fitting, where *r* ^2^ = 1 indicates an ideal linear case. The concentration variation in outlet number 8 could occur from manufacturing defects where the unexpected scratches were transferred to the patterns (Supporting Information Figure S3), leading to the deviation from the linear trendline. Across three experimental replicates, the device provided reliable concentrations with a coefficient of variation (CV) = 2.37 ± 2.35% and a maximum of 9.21% CV. These results demonstrate that the microfluidic device technology can improve the throughput of combinatorial pollutant exposure studies with reliable and reproducible outcomes.

### Morphological Pipeline Assisted by the Combinatorial Microfluidic Dispensation Device Can Describe Cellular Health Profiles Postexposure to Various Real-World Multipollutant Mixtures

To demonstrate the ability of our approach to combine the strengths of morphological and microfluidics-driven multicomponent mixing, we utilized data from our real-world PM characterization in a two-component combinatorial study. We selected manganese (Mn) and selenium (Se) to study the effect of two-component mixtures on lung cell stress response, given the observed variation of these elements within our collected PM samples. We utilized liquid solutions of manganese chloride (MnCl_2_) and sodium selenite (Na_2_SeO_3_) injected into the microfluidic device to perform exposures within a 15-point linear concentration range of 10–150 μM and 1–10 μM, respectively. We selected concentrations that were at least 10-fold greater than those observed from our characterization studies, as we were seeking to evaluate acute cellular responses (24 h liquid exposure). A new UMAP space was created based on the morphological parameters calculated per cell for all exposure conditions across all concentrations (Figure 4A).

**Figure 4:**
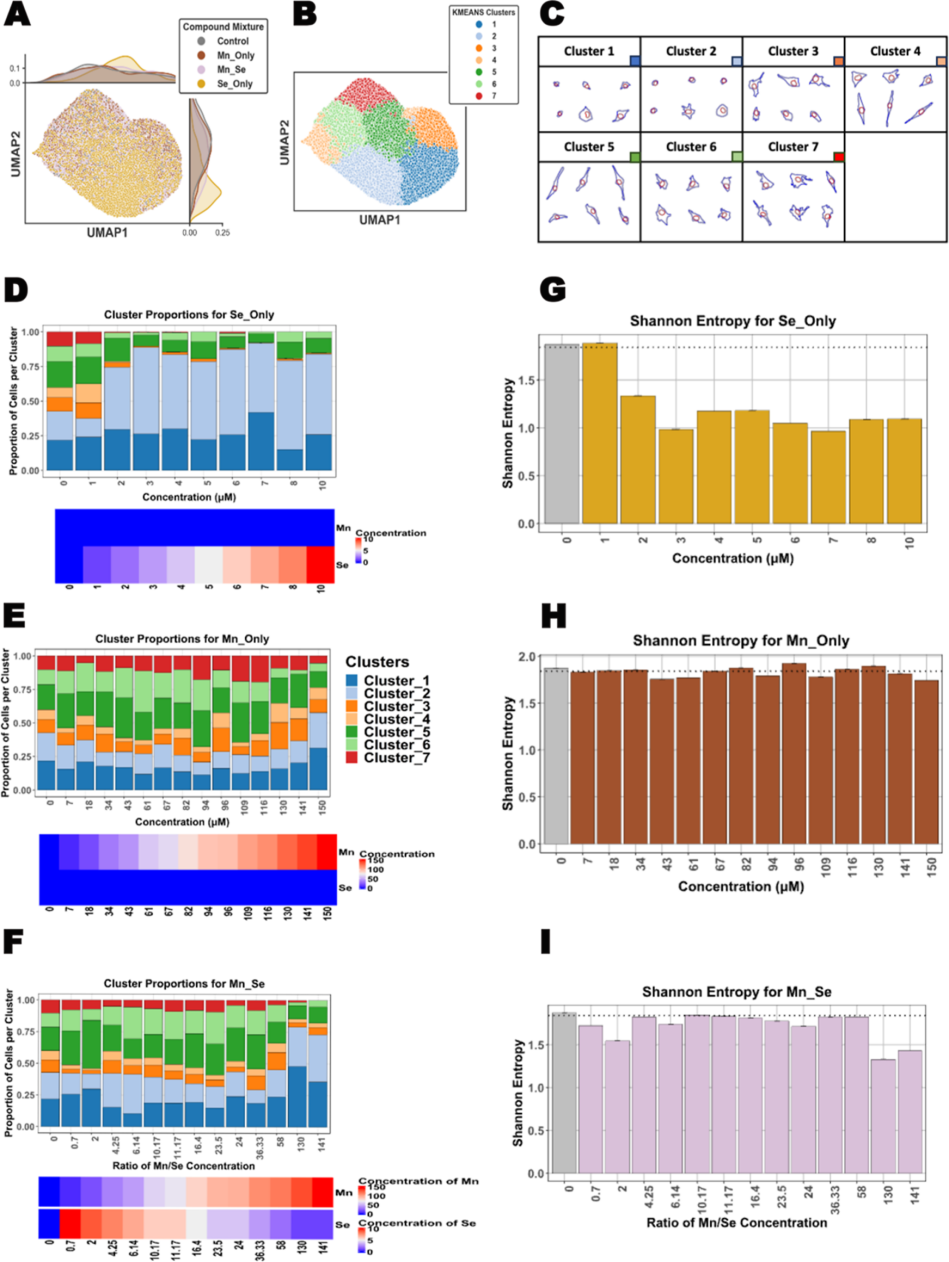
Morphological profiling of lung cells exposed to microfluidics-generated Mn and Se mixtures. (A) Morphological UMAP space of cell populations across conditions and (B) K-means clusters. (C) Morphological clusters generated by control and Mn/Se exposure conditions. Plot of cluster proportions of cell populations exposed to different levels of (D) Se only, (E) Mn only, and (F) Mn–Se mixtures. Shannon entropy plots across (G) Se only, (H) Mn only, and (I) Mn–Se mixture exposures.

Following a similar approach as demonstrated with the formaldehyde single-component analysis (Figure 1A), seven different morphological clusters were extracted from this data set, representing the range of morphological patterns related to the differential responses and cellular health states (Figure 4B,C). From this analysis, we observed dose-dependent shifts in morphological cluster enrichments as a function of exposure conditions (Figure 4D–F). For the Se-only exposures, we observed a sharp shift toward enrichment of clusters 1 and 2 associated with unhealthy phenotypes (small, rounded cells) with increasing concentrations of Se. This enrichment of clusters 1 and 2 was also associated with a concurrent decrease in the remaining clusters 3–7 (Figure 4D). In contrast, even though there was an increase in the enrichment of clusters 1–2 at high concentrations of Mn, clusters 3–4 remained relatively constant across all concentration ranges, along with higher proportions of clusters 5–7 relative to Se only. This result indicates a greater proportion of healthy cell states in the Mn-only exposure condition compared to the Se-only exposure condition (Figure 4E). Interestingly, the drastic enrichment of clusters 1–2 seen with high concentrations of Se was not observed upon the addition of Mn (Figure 4F). This same trend is present when we look at high Mn concentrations with low Se concentration additions, where the enrichment pattern more closely resembles the unexposed (i.e., control) cells (Figure 4F). This result could indicate a decrease in the toxicity of these components at very specific concentration ratios. Cell viability assessment of cells exposed to these mixtures corroborated the same trends, with predetermined mixtures of Mn and Se resulting in more viable cells than the components alone (Supporting Information Figure S4). Shannon entropy calculations to estimate cellular population heterogeneity showed that Mn-only-treated cells retained levels of cellular heterogeneity similar to those of control cells (Figure 4H), while Se-only-treated cells showed decreased heterogeneity in a dose-dependent manner (Figure 4G). This decrease in cellular heterogeneity for Se-exposed cells resulted from the simultaneous depletion of clusters 3–7 and the enrichment of clusters 1 and 2 (Figure 4D). For instance, cells exposed to 2–10 μM Se exhibited a max entropy of less than 1.5, while most of the mixtures with Mn, particularly the ones with a lower Mn/Se ratio, were above that value.

Collectively, these results suggest that morphological profiling can be coupled to high-throughput microfluidic assays and used to detect subtle changes in cell responses from heterogeneous cell populations. These changes can be subsequently used to track the concentration-dependent interactions between multiple chemical compounds.

## Discussion

Here we present a proof-of-concept framework that integrates microfluidic techniques and microscopy-based morphological profiling to detect air-pollutant-driven cellular responses in a high-throughput manner. Developments in morphological profiling have demonstrated the power of utilizing multimodal staining and imaging approaches to successfully predict cell states upon different perturbances, with recent studies showing pipelines that can describe more than 50 different cell health phenotypes.^30^ Here, we utilized a morphological profiling approach and demonstrated that single-cell quantifications based on microscopy can provide a robust assessment of cell health, particularly postexposure to various air pollutant mixtures. Interestingly, we captured distinct morphological states that potentially delineate dynamic transitions between healthy and unhealthy cells (Figure 1), which could provide an additional level of sensitivity and susceptibility not detected by traditional viability assays.

Although air pollution is a major health concern for the general population, evidence shows that specific communities are at increased risk of developing diseases associated with higher exposure to pollutants.^31^ This discrepancy is, in part, due to different geographic areas being more prone to higher air pollution exposure from traffic, industrialization, and other related human activities.^32^ We characterized compositional PM variations in sites around Austin, Texas, that, likely due to geographical distinctions, exhibited differential abundances of several metals (Figure 2). Urban areas, represented by locations A and B, are in close proximity to major highways or high-traffic roads. These locations, as expected, had similar compositional profiles—possessing high concentrations of elements heavily associated with industrial sources (e.g., Mn, Si, Ti).^33,34^ Of significance, a previous study identified high levels of Mn in PM samples obtained from neighborhoods close to major highways and industrial complexes in Houston, TX.^35^ Meanwhile, the same study identified certain elements (e.g., Se) that accumulated more in locations without major sources of emissions, further supporting the results obtained from our collections. Taken together, our results from this pilot study into compositional PM variations across urban and suburban locations identified unique patterns that begin to describe the variability of pollutant exposures within a city like Austin, TX, from a socio-geographical perspective. Future work could expand on this proof-of-concept study to identify compositional patterns associated with a wider array of human activities (e.g., farming, specialized industries, etc.), geographical differences, and seasonal variations to further dissect how these compositional differences lead to disproportionate health outcomes.

The development of biotechnological tools to perform complex cellular and molecular analyses has experienced an exponential growth in the last few decades, facilitating the advancement of many biomedical fields. However, there has been a public call among researchers to start developing tools that could be accessible to communities without the resources for more high-end technologies. This movement toward democratizing science could especially benefit fields such as environmental sciences.^36^ To aid in the systematic assessment of the cellular effects of exposure to different combinations of pollutants, we should strive to create a platform that can accurately generate predetermined mixtures of pollutants within a reasonable time frame, requiring minimal manual labor and low economic burden. In this work, we successfully engineered a microfluidic device to perform multicombinatorial biological exposure studies (Figure 3). We experimentally confirmed the high accuracy and precision of this device, with the potential to perform 15 mixtures of two components per device in just 3 min. While commercial robotic pipettors cost > $20,000, our total device cost is approximately $900 (Supporting Information). Our preliminary combinatorial studies demonstrated that the device is also reusable since the device provided reliable results without contamination for more than 10 trials of experiments. Therefore, given its time-efficient capability, affordability, and reusability, our microfluidic device presents an attractive alternative method to perform multiple combinatorial studies.

Through the observation of morphological patterns, our microfluidics-aided combinatorial study suggests that different ratios of Mn–Se bias cellular responses, with specific combinations decreasing the presence of the unhealthy phenotypes that were observed in single-component exposures (Figure 4). Although Mn is an essential nutrient required by many biochemical processes, Mn excess has been associated with neurotoxicity, lung stress, and the activation of apoptosis in lung cells.^37,38^ Multiple studies have shown that Se supplementation has cytotoxic effects on lung cancer cells, but less information is known about the effect of environmental Se exposure (alone or in combination with other agents) in healthy lung cells.^39^ Our results provide a starting point to evaluate the dynamics of multicomponent acute pollution exposures—utilizing a sensitive, microscopy-mediated assessment of cellular morphology. However, as exposures to air pollutants often occur on a long-term chronic basis, we hope that our pipeline serves as an initial screening tool that could guide focused studies involving chronic exposures to better understand the role of specific pollutant mixtures in lung disease. Moreover, to simplify our pipeline, we utilized a liquid submerged exposure model on immortalized human bronchial epithelial cells (BEAS-2B) to test pollutant mixtures in a high-throughput format. To better mimic the airway cell environment, including cell differentiation processes, future targeted studies could utilize air–liquid interface (ALI) models and primary human bronchial cell lines (as we have done in some of our previous studies^23^) as these can better validate the physiological relevance of the observed cell responses to specific pollutants and their implication in disease.

Taken together, our pipeline leverages a combination of microfluidic, microscopy, and computational technologies to create a sensitive method that measures cell health in a high-throughput format while maintaining a low-cost design. Looking forward, these designs could help empower communities disproportionately affected by air pollution through providing an economical alternative to study the health effects of pollutant mixtures, particularly relevant to specific locations.

## Methods

### Cell Culture and Reagents

Human bronchial epithelial cells (BEAS-2B) were obtained from the American Type Culture Collection (ATCC). Cells were maintained in Airway Epithelial Cell Complete Growth Media (PromoCell) in a humidified environment at 37 °C and 5% CO_2_. All well plates and flasks utilized for cell cultures were precoated with collagen and bovine serum albumin (BSA). For microscopy analysis, cells were seeded on a precoated 96-well plate with a density of 2500 cells/well. For cell viability assays, 8000 cells were seeded on a 96-well plate. Treatments were performed approximately 24 h after cell seeding.

### Formaldehyde Exposure and Imaging

Formaldehyde 16% v/v (ThermoFisher) was diluted to a stock solution of 10 mM in sterile PBS. Live-cell fluorescent labels SPY650-FastAct and SPY555-DNA (Cytoskeleton) were added in a 1:1000 dilution to the growth media for preparing serial dilutions. From the stock solution, serial dilutions ranging from 500 to 31 μM formaldehyde were created using growth media as diluents. Formaldehyde-containing media were then added to the cells and left to incubate at 37 °C for 24 h. After incubation, cells were washed with 1× PBS and then incubated with a 5 μM Calcein Red AM (BioLegend) solution in growth media without phenol red for 30 min at 37 °C. Cells were washed once with PBS, and a final addition of growth media was performed for maintaining cells while imaging. Microscopy images were taken on a Nikon Spinning Disk Confocal Microscope with a 20× ELWD objective and an incubator stage.

### Morphological Analysis

A cell profiler was used to segment images and extract cellular as well as morphological features of the nuclei associated with formaldehyde, Se, Mn, and the combination. Quality control was done to reduce potentially mis-segmented cells and outliers. For construction of the morphological space with formaldehyde, the data was log-transformed as well as standard-scaled and the intensity of Calcein Z-scored across cells. For the morphological space with Mn and Se, as images were captured on different days, we used pyCombat^40^ to standardize the data, estimate, and adjust for potential batch effects that may arise between samples and features, which could confound analyses downstream. pyCombat was initially developed for transcriptomic data, but due to its ability to model multiplicative and additive technical variations with a Bayesian framework, it has since been applied to other high-dimensional data with varying features and samples such as imaging data.^41^ A label of “0” or “Control” was assigned to cells from images where no compound was applied. Post-quality control and normalization, 2512 cells and 93 features were obtained for the formaldehyde condition, and 51,488 cells and 36 features were obtained for the Mn/Se conditions. The morphological features are all derived from the cell profiler measurements (Supporting Information). We hypothesized that these features could help capture the morphological differences that may arise upon exposure to various conditions at various concentrations.

For both the formaldehyde and Mn/Se conditions, we implemented uniform manifold and projection (UMAP), a nonlinear dimensionality reduction technique that allows visualization of our data in a 2-dimensional space where each point represents a cell. Pearson correlation was done to identify highly correlated morphological features that vary along the UMAP space (Supporting Information Figure S5A). To group cells into groups with distinct characteristics, we implemented an unsupervised clustering method called K-means. An optimal number of clusters was selected based on the minimum ratio of the inertia and silhouette values that optimizes the variation between groups of cells while avoiding redundancy across these cluster groupings (Figures 1A, 4A, and Supporting Information S5B).

For investigating how the concentrations of selenium, manganese, and the combination affect the morphological features of the cells, we calculated the proportions of cells in each K-means cluster on a per concentration basis for the Mn-only and Se-only groups (Figure 4D,E). For the Mn and Se combinations, the ratio of Mn to Se was calculated by dividing the Mn concentration by the Se concentration, and the proportion of cells in each cluster per ratio was calculated (Figure 4F). For each compound, we used the control group as a reference, where there was a total concentration of 0 across all compounds. Furthermore, the heterogeneity within each concentration bin across the clusters and conditions was calculated with Shannon entropy (Figure 4G–I). Shannon entropy can be calculated with the natural log as follows

1 $$S_{bin}=-\sum_{i=1}^{7}p_{i}\log\left(p_{i}\right)$$

We calculated a theoretical maximum for the entropy in our data set based on the proportions of all cells in each of the K-means clusters (Figure 4G–I).

### PM Filter Collection

Using a Personal Environmental Monitor (PEM) for PM2.5 sampling, we continuously collected outdoor PM samples on preweighed PTFE filters during 5 days for two different locations at a time, utilizing one location (Location A) as a constant to control for differences related to time of collection. The mass of the PM2.5 collected on filters was measured, and the concentration per volume of collection was calculated. XRF analysis was performed on the samples with an ARL Quant’X EDXRF by the Center for Energy Development and Health at Colorado State University.

### Microfluidic Device Fabrication

The device was patterned on a 4-in silicon substrate, constituting 3 parts: side channels, feed channels, and outlet regions (Figure 3A). We used a mask aligner (Karl Suss MA8, SUSS MicroTec SE) to create a pattern mask onto the substrate and transfer the pattern to the substrate by deep reactive ion etching (DRIE, Versaline DSE, Plasma-therm), as shown in Supporting Information. Figure S8A–D. The first mask layer included the overall structure of the device, and the mask material was positive photoresist AZ5209-E, which was spun at 500 rpm for 1 min, baked at 115 °C for 90 s, exposed at 110 mJ/cm^2^, and developed with AD-10 for 42 s. The second mask layer included the feed channels and outlet regions, and it had negative photoresist SU8-2015 as a mask layer by spin coating at 2000 rpm, soft-baking for 3.5 min, exposing at 145 mJ/cm^2^, hard-baking for 4.5 min, and developing with the SU8-developer for 3.5 min. One mm-diameter outlets were made within these regions by laser drilling (Epilog Laser), as shown in Figure S8E.

After transferring all patterns into the silicon substrate, a Borofloat33 glass wafer with drilled inlet holes and the substrate were cleaned twice with a piranha solution (H_2_SO_4_/H_2_O_2_ = 2:1) for 10 min. A buffered oxide etch (BOE) solution was used to remove native oxide from the silicon substrate. Then, both silicon and glass wafers were bonded together by anodic bonding (AML Wafer Bonder, Applied Microengineering Ltd.), as shown in Figure S8F.

To make connections at inlets and outlets of the device, we bonded a polydimethylsiloxane (PDMS) inlet slab to the top surface of the device and the PDMS outlet slab to the bottom surface of the device (Figure S8G,H). PDMS slabs were cast on a blank silicon mold with a prepolymer to curing agent ratio of 10:1. The PDMS solution was degassed in a vacuum desiccator and cured at room temperature for 24 h. Both inlet and outlet slabs were manually punched, where the holes of the outlet slabs followed the pattern of the outlet locations on the bottom surface of the device. Then, the outlet slabs were plasma-bonded to the bottom side of the device by using manual alignment. A similar plasma bonding procedure was applied for the inlet holes afterward. The poly­(tetrafluoroethylene) (PTFE) tube tips were connected to the outlets to improve the dispensation of the device due to the PTFE hydrophobicity.

## Supplementary Material



## Notes

Additional descriptions and methods for the device fabrication and supplemental experimental data (PDF)
