# Supplementary material for: High-Throughput Microfluidic Technologies for Rapidly Screening Pollutant-Induced Cell Health Effects
Source: ACS Bio Med Chem Au. 2025 Aug 6;5(5):860–9. doi: 10.1021/acsbiomedchemau.5c00094 (PMC12617457; doi:10.1021/acsbiomedchemau.5c00094)
Supplement: Supplementary file 1 [file bg5c00094_si_001.pdf]

Supplementary Information for:

High-throughput microfluidic technologies for rapidly screening pollutant-induced cell health effects

Blanca I. Quiñones-Díaz[a], Niphattha Wongwiset[b], Pratik Kamat[c], Orian Stapleton[d], Sean M. Engels[a], Matthew R. Burroughs[a], S.V. Sreenivasan[b], Jude M. Phillip[c,d,e,f,g], Lydia M. Contreras[a]*

[a]McKetta Department of Chemical Engineering, University of Texas at Austin, Austin, TX 78712, USA
[b]Department of Mechanical Engineering, University of Texas at Austin, Austin, TX 78712, USA
[c]Department of Chemical and Biomolecular Engineering, Johns Hopkins University, Baltimore, MD 21218, USA
[d]Department of Biomedical Engineering, Johns Hopkins University, Baltimore, MD 21218, USA
[e]Department of Oncology, Sidney Kimmel Comprehensive Cancer Center, Johns Hopkins University, Baltimore, MD, 21287
[f]Translational Tissue Engineering Center (TTEC), Johns Hopkins University, Baltimore, MD 21287, USA
[g]Institute for Nanobiotechnology (INBT), Johns Hopkins University, Baltimore, MD 21218, USA

*Corresponding author: lcontrer@che.utexas.edu

Supporting Text:

SI: The design of pre-determined concentrations by side-channel geometries of the microfluidic device

The outlet flow ratios within each inlet branch were designed using hydraulic-circuit analogy, $\Delta P = QR_{hyd}$, where ΔP is pressure drop, Q is constant flow rate, and R_{hyd} is hydraulic resistance[1]. With the height difference between the feed and side channels at 10x, as listed in SI - Geometry section, the resistances of feed-channel and outlet regions were considered insignificant, so all side-channel resistances were connected in parallel (Fig. S6-A). The resistances of feed channel and outlet regions are 17x and 380x less than the smallest side-channel resistance. The flow ratios were calculated from Eq. 1 where r_s is the outlet flow ratio at a specific outlet, q_s is the outlet flow rate at the outlet, q_i is the flow rate into the inlet branch, R_s is the side-channel resistance at the outlet, and R_{tot} is the total resistance of the inlet branch calculated from Eq. 2. R_{end} is the end-side channel resistances; n is the total number of the end-side channels; and i represents an individual side channel where its inlet branch has m total side channels.

$$r_s = \frac{q_s}{q_i} = \frac{R_{tot}}{R_s} \quad (1)$$

$$R_{tot} = \left(\sum_{i=1}^{m} \frac{1}{R_s(i)} + \frac{n}{R_{end}}\right)^{-1} \quad (2)$$

Based on the computation of Eqs. 1 and 2, the flow ratios were designed from the geometries of the side channels. In this design, the side channels had parallel rectangular shapes with 400-μm pitch and alternating gaps to create a linear gradient concentration (Fig. 3C). The resistances of the parallel rectangular channels ($R_{parRect}$) were calculated from Eq. 3, where μ is liquid dynamic viscosity, p is the pitch of the parallel channel, t is the stripe thickness of the channel, L is channel length, W is channel width, and h is channel height; all referred geometries are also included in SI-Geometry section. R_{end} is at least 70x more than R_s in this design to minimize waste volume.

$$R_{parRect} = \frac{12\mu L}{\max(p-t,h)\,\min(p-t,h)^3}\left(1 - 0.63\,\frac{\min(p-t,h)}{\max(p-t,h)}\right)^{-1}\left\lfloor\frac{W}{p}\right\rfloor \quad (3)$$

SI: Summary of the geometry of the microfluidic device

The following information in this section is the geometric configuration of the experimented microfluidic device.

Feed-channel width	=	2,000	μm
Feed-channel total length	=	130,250	μm
Feed-channel height	=	289.90 ± 9.38	μm
Outlet-region height	=	289.90 ± 9.38	μm
Side-channel width (W)	=	2,000	μm
Side-channel height[2]	=	29.83 ± 1.11	μm
Side-channel length (L)	=	2,400	μm
Side-channel pitch (p)	=	400	μm
Well-spacing	=	9,000	μm

SI: Cost computation of the microfluidic device

The cost computation comprised 3 parts: (1) the device fabrication cost in Table S-3, (2) the device material cost in Table S-4, and (3) the instrumental cost. The total fabrication and material cost per wafer, which constitutes two identical devices, was \$416, resulting in a per-device cost of \$208. The total setting cost is \$908 comprising \$208 for the device and \$700 for instrumental from two syringe pumps.

SI: Morphological parameters used to cluster formaldehyde-exposed cells

AreaShape_Area	AreaShape_BoundingBoxArea	AreaShape_Compactness
AreaShape_Eccentricity	AreaShape_EquivalentDiameter	AreaShape_EulerNumber
AreaShape_Extent	AreaShape_FormFactor	AreaShape_MajorAxisLength
AreaShape_MaxFeretDiameter	AreaShape_MaximumRadius	AreaShape_MeanRadius
AreaShape_MedianRadius	AreaShape_MinFeretDiameter	AreaShape_MinorAxisLength
AreaShape_Perimeter	AreaShape_Solidity	Nuclei_AreaShape_Area
Nuclei_AreaShape_BoundingBoxArea	Nuclei_AreaShape_Compactness	Nuclei_AreaShape_Eccentricity
Nuclei_AreaShape_EquivalentDiameter	Nuclei_AreaShape_Extent	Nuclei_AreaShape_FormFactor
Nuclei_AreaShape_MajorAxisLength	Nuclei_AreaShape_MaxFeretDiameter	Nuclei_AreaShape_MaximumRadius
Nuclei_AreaShape_MeanRadius	Nuclei_AreaShape_MedianRadius	Nuclei_AreaShape_MinFeretDiameter
Nuclei_AreaShape_MinorAxisLength	Nuclei_AreaShape_Perimeter	

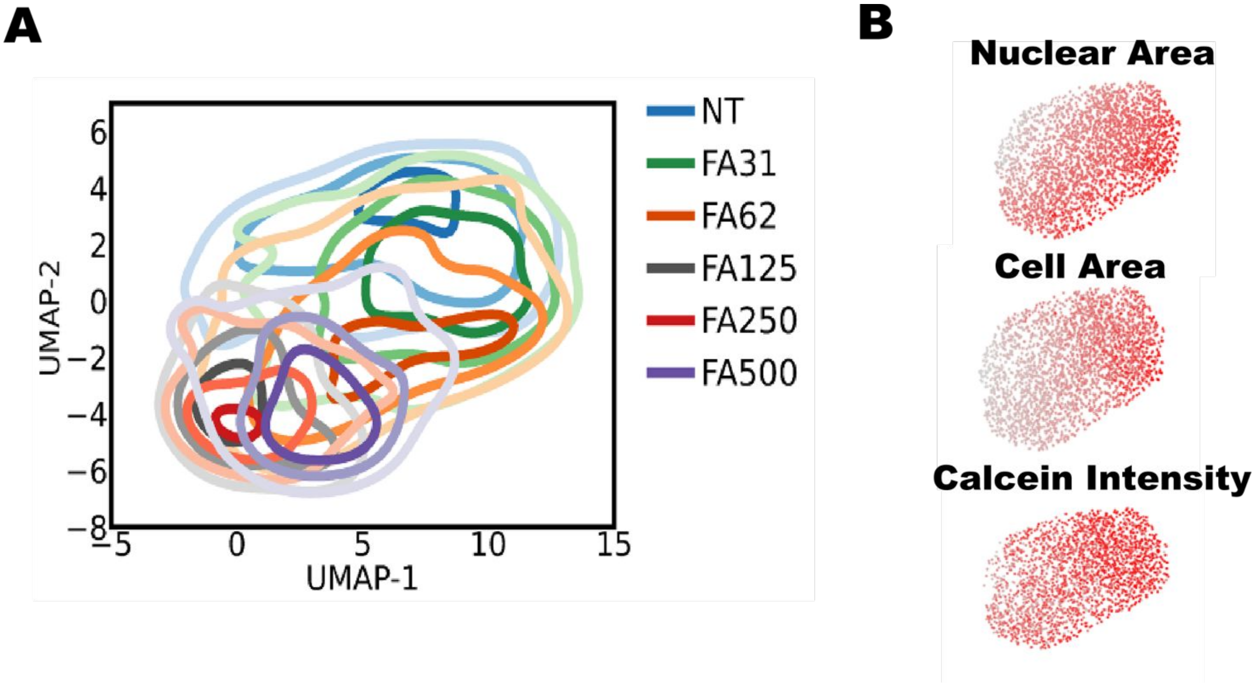

Figure S1. (A) Contour map of formaldehyde exposures in UMAP space. **(B)** Feature intensity map of nuclear area, cell area and calcein intensity in UMAP space.

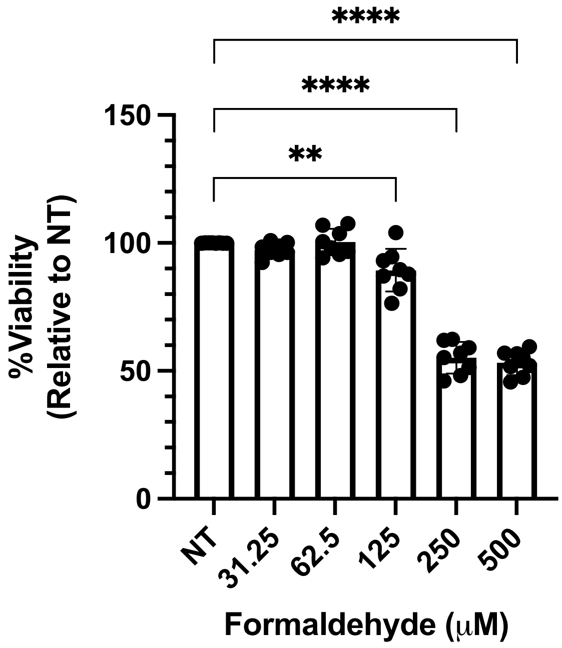

Figure S2. Cell viability as measured by AlamarBlue assay of cells exposed to formaldehyde for 24-hrs. One-way ANOVA test was performed to determine statistical significance (**p-value<0.01, ****p-value<0.0001).

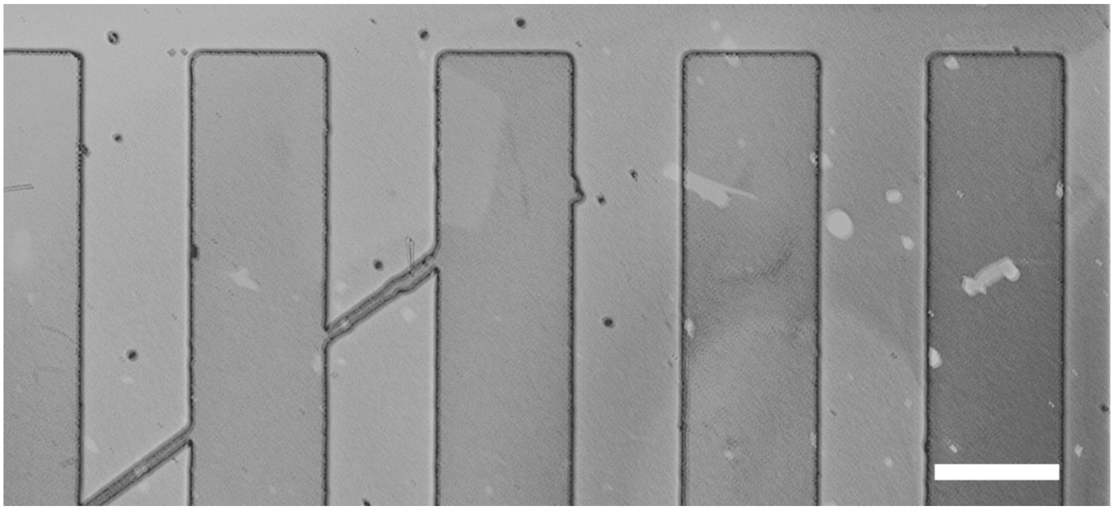

Figure S3: Microscopic picture of unexpected scratch patterns at outlet number 8 of Branch B. This pattern partially blocked liquid in the side channel of the microfluidic device. Scale bar is 100 μm.

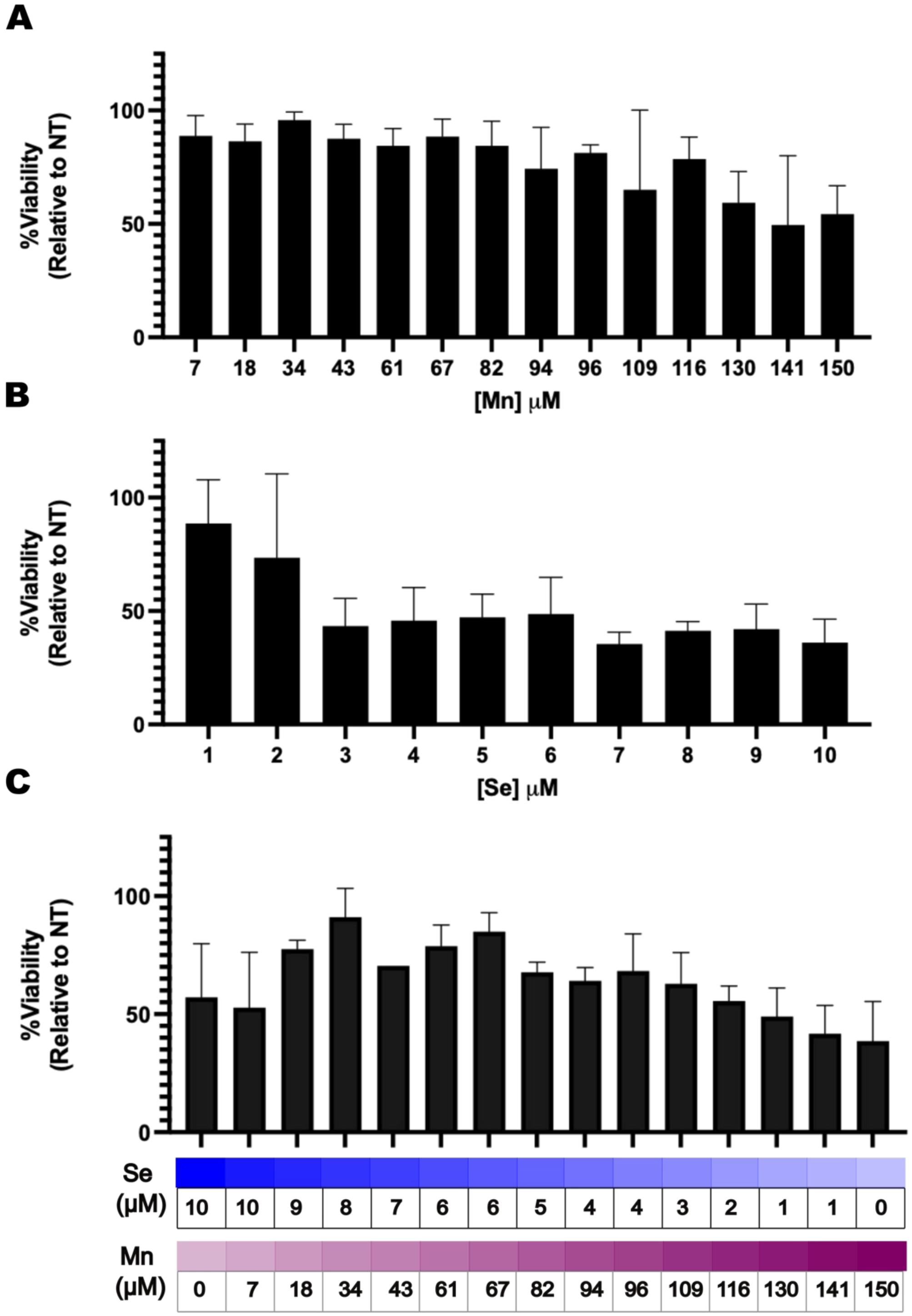

Figure S4. Cell viability normalized to non-treated (NT) cells for **(A)** Mn only **(B)** Se only and **(C)** Mn-Se mixtures exposures.

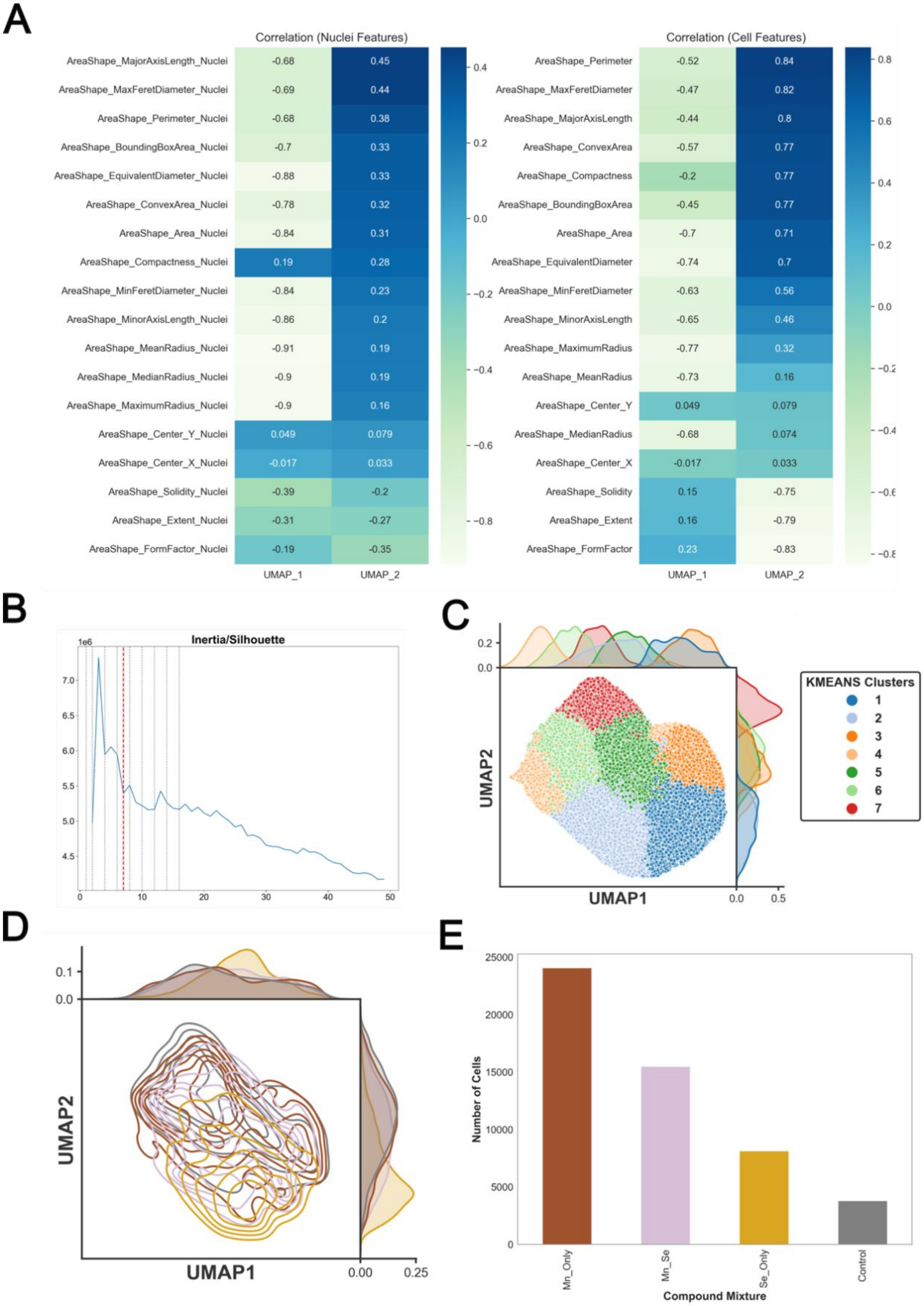

Figure S5. (A) Pearson correlation for nuclei and cell features. **(B)** Ratio of the inertia and silhouette values between group of cells. **(C)** UMAP clustering representation. **(D)** Contour map of UMAP space across all Mn and Se conditions. **(E)** Plot of number of cells analyzed for each condition.

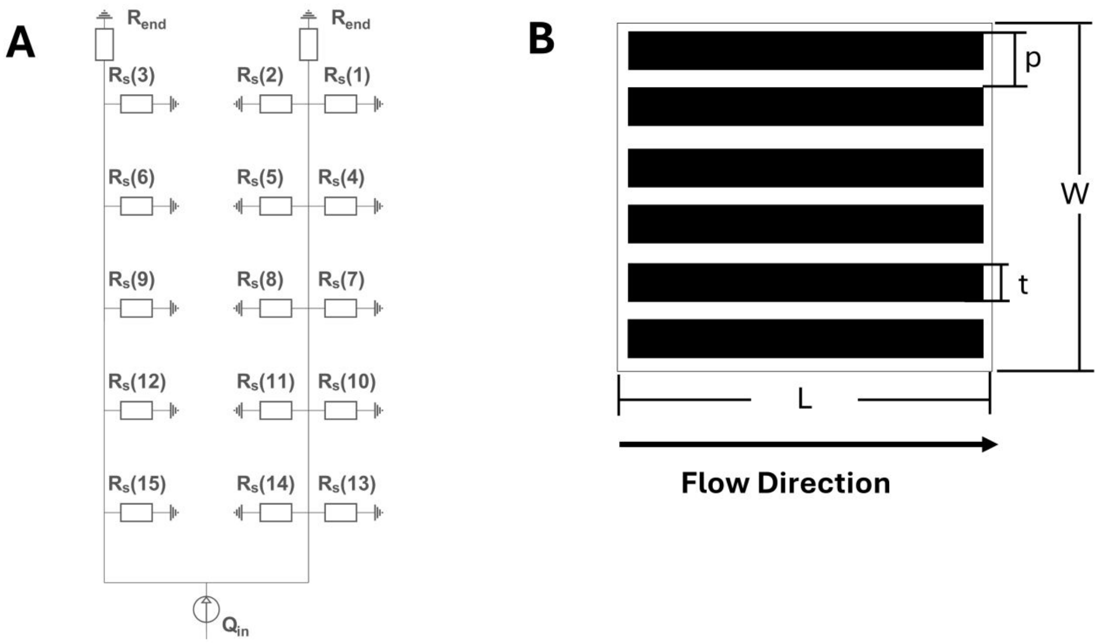

Figure S6: (A) microfluidic circuit network of Branch B. Note that branch A has a similar circuit network with180-degree, clockwise orientation. **(B)** side-channel geometry for resistance computation.

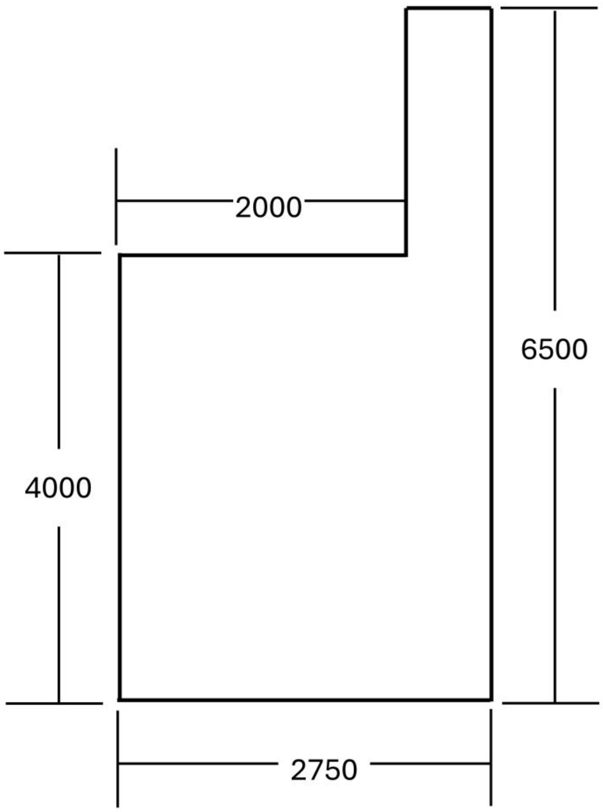

Figure S7: The geometry of the outlet region. The unit is in microns.

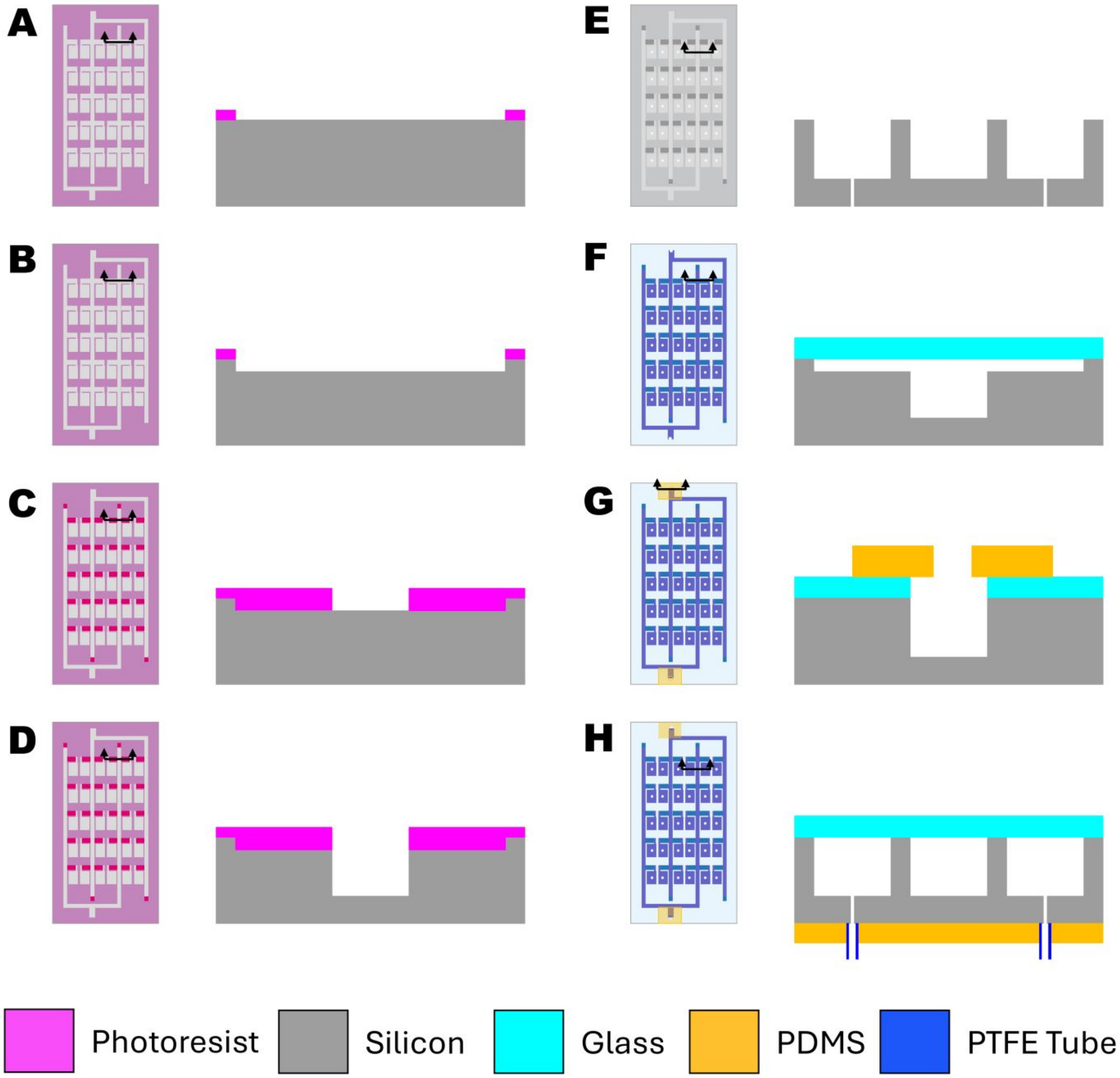

Figure S8: Manufacturing schematics of the microfluidic device. **(A)** The first mask including all structures was patterned on a silicon substrate by lithography. **(B)** The first pattern was transferred into the substrate by DRIE. **(C)** The second mask consisting of feed channels and outlet regions was patterned on the substrate by lithography, **(D)** The second pattern was transferred into the substrate by DRIE to deepen the regions. **(E)** The substrate was laser drilled on the outlet regions to create through holes. **(F)** The glass wafer with drilled inlet holes and the substrate were anodically bonded to enclose the channels. **(G)** Two PDMS slabs were plasma bonded at the inlets for inlet-tube connections. **(H)** The outlet PDMS slab was aligned to the outlets of the device and then plasma bonded. To enhance dispensation, PTFE tubes were inserted at all outlets.

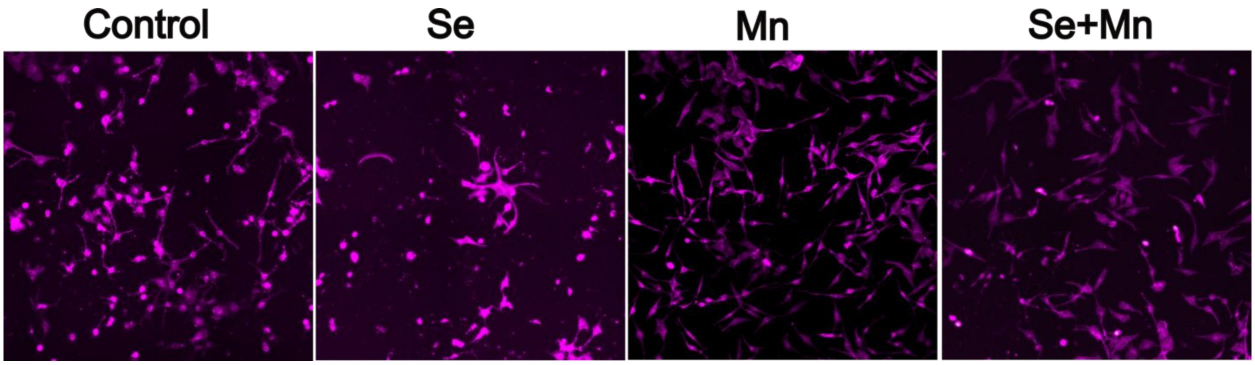

Figure S9. Representative images of BEAS-2B cells under control, Se (6μM), Mn (94μM) or Se-Mn mix.

Table S1: Concentration of elements in PM samples (ng/μg total PM)

Element	Week 1		Week 2		Week 3	
	Location A	Location B	Location A	Location C	Location A	Location D
Mg	8.5628	7.3078	6.9140	5.7967	7.2189	8.2052
Al	5.6456	5.6059	18.2847	18.8862	11.9788	8.7724
Si	20.7080	18.4795	54.5960	50.4580	46.9513	30.9887
P	0.2468	0.2296	0.4644	0.5489	0.3405	0.2455
S	37.9099	36.5392	39.7225	47.8466	34.8203	37.0746
K	15.5119	16.4739	14.1155	13.7068	12.1996	11.3545
Ca	43.1195	38.6163	49.8686	27.6890	110.1996	77.7237
Ti	1.1012	0.7638	1.5411	1.3906	1.7488	1.0448
Mn	0.1856	0.1496	0.3265	0.2111	0.3474	0.2660
Fe	10.2843	8.2325	16.2189	11.6438	15.2137	11.7462
Zn	1.3440	1.4115	0.9042	0.8482	1.1400	1.2724
Se	0.3407	0.3064	0.2249	0.2960	0.1788	0.2078
Sr	0.1097	0.0893	0.2407	0.1276	0.2901	0.1522
Pb	0.8181	0.8532	0.7539	0.7894	0.6506	0.6791

Table S2: Microfluidic device stripe thicknesses of the side channel (t) where i, A, B represent outlet index at Branches A and B, respectively.

Outlet index (i)	$t_{i,A}$ (μm)	$t_{i,B}$ (μm)
1	-	28
2	365	54
3	339	80
4	313	106
5	287	132
6	261	158
7	235	183
8	209	209
9	183	235
10	158	261
11	132	287
12	106	313
13	80	339
14	54	365
15	28	-
End	385	385

Table S3: Fabrication cost of the microfluidic devices per wafer. The estimated cost was computed from the tooling fees (C_t) at the Microelectronic Research Center of the University of Texas with the labor cost (C_l) at $28/hour.

**Manufacturing Process**	**Processing time (hour)**	**Tooling fee/hour (C_t)**	**Manufacturing cost ($C_m = t \times (C_l + C_t)$)**
**Layer 1: Side channel**			
Spin coating	0.083	$ 10.00	$ 3.17
Lithography	0.083	$ 50.00	$ 6.50
Etching	0.167	$ 50.00	$ 13.00
Piranha clean	0.500	$ 10.00	$ 19.00
**Later 2: Feed channel and damper**			
Spin coating	0.17	$ 10.00	$ 6.33
Lithography	0.08	$ 50.00	$ 6.50
Etching	0.33	$ 50.00	$ 26.00
Piranha clean	0.50	$ 10.00	$ 19.00
**Laser drilling of thorough holes**	0.17	$ 50.00	$ 13.00
**Piranha clean**	0.50	$ 10.00	$ 19.00
**Anodic bonding**	2.00	$ 50.00	$ 156.00
**PDMS bonding**	0.50	$ 50.00	$ 39.00
**Total**			**$ 326.50**

Table S4: Material cost (C_{mat}) per wafer of the microfluidic device

**Materials**	**Cost (C_{mat})**
Silicon wafer	$ 20.28
Glass wafer	$ 20.28
Photoresist	$ 4.23
PDMS	$ 20.02
Blunt needles	$ 1.02
Syringes	$ 3.04
Filter	$ 10.08
PTFE tubes and tips	$ 10.12
**Total**	**$ 89.07**

Reference

1 H. Bruus, *Theoretical Microfluidics*, Oxford University Press, 2009.
